# Perinatal Predictors of Postpartum Depression: Results of a Retrospective Comparative Study

**DOI:** 10.3390/jcm9092952

**Published:** 2020-09-12

**Authors:** Jolanta Banasiewicz, Kornelia Zaręba, Małgorzata Bińkowska, Hanna Rozenek, Stanisław Wójtowicz, Grzegorz Jakiel

**Affiliations:** 1 Department of Medical Psychology and Medical Communication, Medical University of Warsaw, 00-575 Warsaw, Poland; jolantabanasiewicz.wum@gmail.com (J.B.); hanna.rozenek@wum.edu.pl (H.R.); stwojt@o2.pl (S.W.); 2 Department of Obstetrics and Gynecology, Center of Postgraduate Medical Education, 01-004 Warsaw, Poland; grzegorz.jakiel1@o2.pl; 3 Department of Gynecologic Oncology and Obstetrics, Center of Postgraduate Medical Education, 00-416 Warsaw, Poland; mabi@onet.pl

**Keywords:** baby blues, postpartum depression, postpartum mood disorders, perinatal predictors of depression, EDPS

## Abstract

Postpartum mood disorders occur in a considerable number of women with the most common postpartum disorder being baby blues. The study aimed at the identification of the risk factors present before delivery, which may be comprised in prophylactic programs concerning postpartum mood disorders. The research material includes data retrieved from the medical record of patients delivering in Warsaw in the years 2010–2017 who routinely completed Edinburgh Postnatal Depression Scale (EPDS) after delivery. Data of 604 patients were analyzed. The study group included 75 women who obtained at least 12 points in EPDS, which constituted 12.4% of the whole group (mean = 14.92, SD = 3.05). The control group was made up of 75 women who obtained no more than 5 points in EPDS. A significant correlation was reported between the parity and their order vs. the risk of developing postpartum mood disorders. Women with an increased risk delivered at about 37 gestational weeks, while women in whom the risk of such disorders was low delivered at about 39 gestational weeks. No increased risk was noted in women with premature rupture of membranes. Primigravidas and women who delivered prematurely were the most predisposed to developing postpartum depression and should undergo screening tests in the perinatal period.

## 1. Introduction

The interest of researchers regarding mental disorders occurring in women in labor mostly focused on issues associated with postpartum depression. However, statistical data analysis showed that baby blues was the most common disorder affecting women after delivery [1]. According to the Diagnostic and Statistical Manual of Mental Disorders, Fifth Edition, issued by the American Psychiatric Association (APA), such a phenomenon was referred to as adjustment reaction with depressed mood [2,3]. Baby blues most commonly occurs in developed countries (55–85%). Subsequently, over 20% of women may develop postpartum depression [4,5]. Postpartum depression occurs at various frequencies, depending on the type of test, cut-off values of depression measurement scale and the country where testing is conducted. Its prevalence may even reach 39% of women in some countries, e.g., Bangladesh [6]. Psychotic disorders were reported much less frequently [2]. Baby blues constitutes a prelude to subsequent more severe psychiatric conditions, so it is of utmost importance to introduce early diagnostics and distinguish the group at risk.

According to some authors, postpartum mood depression is a physiological side effect of a woman developing into a mother [7]. Baby blues is a disorder characterized by a mild course. It is limited to the first weeks after delivery. The first symptoms appear about 3–4 days after delivery and persist for up to several weeks. The most common symptoms of baby blues include mood worsening, tearfulness, emotional lability, irritability, increased susceptibility to frustration, headaches, concentration problems, anorexia, and sleep disturbances [8]. The symptoms usually resolve within 6–9 weeks postpartum.

The etiology of baby blues has not been fully elucidated. Its causes may be related to biological, psychological and environmental factors [1,9]. As regards the biological factors, the most important role is attributed to hormonal changes in a woman’s body after delivery. Estrogen and progesterone levels are decreased while prolactin level is increased [10]. The concentrations of progesterone, estradiol and free estriol decrease by approx. 90–95% over a few days after delivery [11,12]. A key role is also attributed to physiological pain during the postpartum period which is associated with the healing of perineal or abdominal wound, shrinking of the uterus, breast pain linked to lactation, or spinal pain related to the change in body statics in women after delivery. As regards the psychological factors, the following ones are of key importance: mental tendencies, especially a history of anxiety and depressive disorders [1,13,14]. A history of PMS and mood changes attributable to menstrual cycles are significant predictive factors [15,16,17,18]. Fear about the health and life of the child and the feeling of uncertainty associated with changes to happen in life also play a major role [14,19]. Moreover, in the contemporary world, it is also emphasized that the reduced attractiveness and increased body weight compared to the pregestational period contribute to postpartum mood disorders [8]. Some authors reported that depressed mood was also associated with the discomfort perceived by women resulting from “the loss of freedom” and independence [7].

The majority of currently available studies did not reveal a statistically significant relationship between postpartum mood depression and demographic variables. No correlation was observed between the age, marital status, socioeconomic status, environmental stimuli and the risk of developing postpartum mood disorders [12,20].

Correlations between postpartum mood worsening and data concerning obstetric and gynecological history have not been fully elucidated as well. According to some authors, primiparas were more prone to postpartum mood depression than women who had already given birth. However, others claimed that multiparas developed the disorder more commonly [8].

The review of the results of studies conducted so far reveals a lot of ambiguity. Notably, the abovementioned studies were conducted over a variety of time perspectives and socio-cultural settings. Therefore, we attempted to identify risk factors present before delivery, which may be comprised in prophylactic programs concerning postpartum mood disorders.

## 2. Material and Method

### 2.1. The Aim of the Study

The study aimed at the identification of the predictors of mood worsening in women during the first week postpartum. The following research questions were proposed:

- What demographic variables are correlated with the risk of developing depressive disorders after delivery?
- What variables in the obstetric and gynecological history are correlated with the risk of developing depressive disorders after delivery?
- What anti-health behaviors (tobacco smoking) declared by women are associated with the risk of developing depressive disorders after delivery?
- Are psychological problems declared by women before and during pregnancy associated with the risk of developing depressive disorders after delivery?

### 2.2. Material

It is a retrospective comparative study. Study material includes data retrieved from medical records of patients hospitalized in the Teaching Department of Gynecology and Obstetrics of Prof. Orłowski Hospital in Warsaw in the years 2010–2017. The medical records were analyzed in terms of selected data, i.e., demographic variables, variables concerning the general health status, variables concerning obstetric and gynecological history. EPDS scale is routinely attached to the medical records of the Obstetric Department of the hospital. Women in labor who are hospitalized in the Department are asked to complete the questionnaire as part of screening testing 2–4 days after delivery. The aim of asking patients to complete EPDS was to obtain information concerning the current frame of mind of postpartum patients and, if necessary, to provide a suitable form of psychological or psychiatric support.

Only patients from whom informed consent could be obtained were qualified for EPDS test. The consent was obtained orally and confirmed via voluntary completion of the questionnaire. The exclusion criterion for the EPDS test was no knowledge of Polish, which prevented questionnaire completion. All subjects gave their informed consent for inclusion before they participated in the study. The study was conducted in accordance with the Declaration of Helsinki, and the protocol was approved by the Ethics Committee of the Center of Postgraduate Medical Education in Warsaw research number 63/PB/2017.

The control group was included in the study. Pairwise selection method was implemented. The criteria of assigning women to the control group were age, place of residence, marital status and the EPDS score below 5. Data of 604 patients were analyzed. The study group included 75 women who obtained at least 12 points in EPDS, which constituted 12.41% of the whole group (mean = 14.92, SD = 3.05). The control group was made up of 75 women who obtained no more than 5 points in EPDS.

### 2.3. Research Tool Description

Edinburgh Postnatal Depression Scale (EPDS) is most commonly used as a screening test in the risk assessment of developing depressive disorders. It was authored by Cox, Holden and Sagowski [21,22]. We used the Polish version of the scale translated by Maria Bnińska, MSc, PhD [23]. The authors of EPDS and the British Journal of Psychiatry, being the owners of the copyright, accept the use and copying of the tool provided that the source is cited [21]. The scale is used for the assessment of the frame of mind over the last week prior to the test. EPDS is a short scale. It comprises 10 statements describing various aspects of the frame of mind of women, such as anhedonia, sense of guilt, anxiety, panic attacks, exhaustion/overtiredness, sleep disturbance, sadness/dejection, tearfulness and suicidal thoughts. The total of all obtained points means the general score (max. 30 points). The higher the score, the higher the index of the risk of developing postpartum depression in the tested person. The borderline value indicating an increased risk of depression was assumed to be 12/13 points with the reservation that some specialists recommended vigilance even in case of scores lower by several points [22,24]. Moreover, particular attention is necessary in cases when the patient reported the presence of intense suicidal ideation, even if the total EPDS was low [21].

EPDS is characterized by good psychometric properties. According to original research, the sensitivity of the tool was 86%, the specificity was 78%, and the Cronbach’s alpha coefficient was 0.88 [22].

### 2.4. Statistical Analysis

Pairwise selection method was used to assess the risk of developing postpartum mood depression. The criterion variables were age, level of education and the marital status. We assumed the lowest *p* value at 0.05. Logistic regression analysis was performed, but it showed no significant risk factors, so its results were not presented in the paper. No tested variables presented normal distribution (Kolmogorov–Smirnov test). Therefore, comparative analyses between the groups were performed with the use of non-parametric tests: the Mann–Whitney U test for variables measured with ratio scales and the chi-square test for nominal variables. The study was conducted with the use of IBM SPSS Statistics for Windows, v24.0., Armonk, NY, USA: IBM Corp. (released 2016).

## 3. Results

### 3.1. Demographic Data

Study group patients were aged 18–45 with the average age being 30.6 years (SD = 4.70). The majority of patients completed tertiary education (66%). Most of them were married (83%) and over half lived in a city (54.7%) (Table 1). No significant differences were revealed between the place of residence and the risk of developing depressive disorders after delivery (F = 0.339; *p* = 0.713). Other demographic parameters were not tested, because they were not the enrollment criteria for the control group.

### 3.2. Current Pregnancy History

A statistically significant correlation was reported between pregnancy duration and the risk of developing postpartum depressive disorders. The shorter the pregnancy duration, the higher the risk of developing postpartum depressive disorders was noted. Women with an increased risk of developing postpartum depressive disorders delivered at about 37 gestational weeks, while women in whom the risk of such disorders was low delivered at about 39 gestational weeks (Table 2). A significant correlation was reported between the parity and the order vs. the risk of developing postpartum depressive disorders.

Premature rupture of membranes (PROM) was reported in 18 women in whom the risk of postpartum mood disorders was high and occurred slightly more commonly than in the control group (*N* = 17) (Table 3). However, no statistically significant intergroup differences were noted.

Diseases and congenital defects diagnosed in the child intragestationally were reported in 33 mothers from the group at a high risk of developing postpartum mood disorders. In this group, children were affected by defects and diseases more commonly than in the control group (*N* = 23). However, no statistically significant intergroup differences were noted (Table 4).

Psychological problems before and during pregnancy were reported by a low number of participants (8), so no results of statistical analyses are available. Intragestational mental problems were not reported by any participant.

The Mann–Whitney U non-parametric analysis revealed no statistical significance as regards the influence of tobacco smoking on the risk of developing postpartum depressive disorders (Table 5).

## 4. Discussion

Irvin Yalom—one of the best known American psychiatrists and psychotherapists—claimed that postpartum mood depression was a typical physiological reaction to physical stress associated with pain, tiredness, hormonal changes, and mental stress related to a new situation and role in one’s life [25]. However, the present study and other authors demonstrated that variables present before delivery were significant risk factors of postpartum depression.

A study conducted in China showed that exposure to cellular phones during pregnancy, maternal age and the gestational age at which the pregnancy was completed were positively correlated with postpartum depression [26]. Young mothers more commonly felt incompetent and fearful about the child compared to older mothers. A population-based study showed a positive correlation between PPD and factors like older age (RR 1.25). The same study showed that the risk of PPD decreased after the first month postpartum in young women (RR 2.14), those who underwent instrumented delivery (RR 1.23) or Cesarean section (RR 1.64) and in case of moderate preterm delivery (RR 1.36) [27]. Maharjan et al. conducted a study in Nepal. They reported ethnicity and age at which the woman got married to influence the development of postpartum depression [28]. They also did not report a relationship between an increased risk of postpartum depression and the level of education, family income, tobacco smoking, and the duration of marriage [28]. Similarly, the present study showed that tobacco smoking did not affect the risk of developing postpartum depressive disorders.

According to Sun et al., a higher prevalence of postpartum depression was noted in primiparas (20.3–29%) compared to multiparas 13.5% [29]. Similar results were obtained by Ly Do who demonstrated a twofold increase in the risk in primiparas [30]. According to Maharjan et al. who conducted a study in Nepal, the development of postpartum depression was affected by the number of children, the sex of the child who was born and the planned or non-planned character of the pregnancy [28]. A study carried out in Nigeria revealed that a high number of children alive was a protective factor as regards PPD [31]. The present study demonstrated a higher risk of mood disorders in primiparas than in multiparas. A higher intensity of anxiety in primiparas may be related to the feeling of incompetence and fear of the new, unknown experience [32]. The results of previous research conducted in this area are not consistent. Mathisen examined 86 Argentinian women who had delivered in a private hospital in Buenos Aires and demonstrated that the risk of developing postpartum mood disorders increased with parity [33]. The specificity of the place, i.e., a private hospital, in which the study was conducted, might influence the results. A study conducted in 13 Japanese hospitals in a group of 3769 women in labor revealed that the risk of developing postpartum mood disorders was higher in primiparas than in multiparas over the first month postpartum. However, the difference regressed in subsequent months after delivery [34].

This study showed that shorter pregnancy duration translated into a higher risk of depressive disorders after delivery. Available research results confirmed this rule. Shorter pregnancy duration increased the risk of postpartum mood disorders. As a result of preterm delivery, neonates had the features of prematurity, which made them more susceptible to infections and necessitated future treatment, which increased fear and anxiety in the mother [35]. The study of Vigod showed that mood disorders occurred in approx. 40% of women who delivered prematurely [35]. Cherry et al. conducted a study in a group of American women in labor who delivered prematurely and whose neonates stayed in intensive care units. The risk of postpartum mood disorders was reported in over 36% of those mothers [36].

A cross-sectional qualitative study was conducted in Vietnam. It showed that the increased risk of PPD was correlated with the level of education (low level of education), intragestational diseases (diabetes, hypertension, liver pathologies, infections) and the lack of satisfaction with family life. However, the limitation of communication and interaction with the environment constituted the strongest predictors of PPD (*p* < 0.0001, OR 4.4.) [30]. Women who declared to be happy after delivery and satisfied with family life were characterized by a less frequent occurrence of PPD symptoms. A population-based study showed a positive correlation between PPD and such factors as gestational diabetes mellitus (RR 1.7), pregestational depression (RR 1.49), pregestational diabetes (RR 1.49) and mild preterm delivery (RR 1.20) [27]. Maternal hypertension was found to increase the risk of postpartum depression [26,27]. The risk increased due to preterm delivery, delayed initiation of breastfeeding and lower birth weight of the child. A retrospective study by Chen et al. demonstrated that the risk of postpartum depression was 3-fold higher in women who experienced preeclampsia intragestationally, whilst in case of severe preeclampsia, the risk was even 4-fold [37]. Similarly, pregestational obesity also indirectly correlated with the risk of PPD, probably due to the obesity-induced stress mechanism [38]. According to anthropometric studies, the increased prevalence of mood disorders was due to body weight, height, body weight gain during pregnancy, BMI and waist-to-hip ratio, which also mostly contributed to the percentage of suicides after delivery and the severity of depression [39]. Amirchaghmaghi studied the occurrence of postpartum depression in couples who conceived via assisted reproductive technology (ART). The percentage of postpartum depression cases was lower in couples who conceived via ART compared to those who conceived naturally (20.4% vs. 26%) [40]. The present study also showed no increase in the risk of postpartum depression in women after fertility treatment.

Low socioeconomic status, being a widow, poor social support and death in the closest family positively correlated with the risk of postpartum depression in a study conducted in 308 women in Ethiopia [41]. The authors emphasized the significance of being married as a protective factor of postpartum depression. A study conducted in the Bronx showed that unemployed women were twice more commonly diagnosed with PPD (27.04%) compared to women who worked (13.95%) [26]. Chen et al. conducted a study in immigrants in Taiwan. They demonstrated that women characterized by lower domestic-decision-making power and social support during pregnancy were more commonly diagnosed with postpartum depression. Similar results were obtained in women working full-time and with lower family income [37]. The authors suggested that the population of immigrants was healthier and more motivated, so depression occurred less frequently than in the local population. A study conducted in Nepal by Maharjan et al. revealed the percentage of postpartum depression of 15.2%. The occurrence of postpartum depression was influenced by ethnicity, poor relationship with parents-in-law, and alcohol drinking in the partner. No differences were noted as regards family financial status [28]. A study carried out in Nigeria revealed a positive correlation between poor social support, multiparity and psychological distress after delivery or postpartum depression [31]. The symptoms of anxiety (30.1%) and depression (33.3%) were very high within 14 days postpartum. A study conducted in 300 women in Iran showed that the risk factors of postpartum depression included physical function (*p* = 0.32), role impairment due to emotional health (*p* = 0.02), fatigue (*p* = 0.02) and social function (*p* = 0.002) [42]. The most outstanding risk factors of depression were: poor family support (7%), anxiety about the newborn (6%), a history of depression (2%), relationship problems (3%), financial problems (3%), lack of relaxation (8%) and a combination of all factors (72%). Pre- or intragestational mental problems were not reported in any woman from the study or the control group of the present study. It may be due to the stigmatization of mental diseases in Polish society, which may contribute to the fact that the patients did not report them during conventional history taking. The team frequently learned that patients had a history of psychiatric disorders only when psychotic symptoms occurred after delivery.

The mental status and frame of mind of a woman after delivery is highly significant for the entirety of the physical and mental health of the child and the functioning of the whole family. Robakis et al. demonstrated that cautious antenatal optimism outlook on motherhood was the strongest protective factor of postpartum depression [43]. Medical personnel may stand the chance of strengthening this feature from the very beginning of the care of a woman in labor by suggesting relaxation methods or psychological support. The identification of patients at a higher risk of postpartum depression is crucial as early as the perinatal stage. Numerous pharmacotherapy limitations have to be regarded in pregnant women. However, there are various forms of psychotherapy, such as cognitive behavioral therapy, mindfulness, interpersonal therapy and the increasing potential of telemedicine, which are completely safe in pregnancy [44]. Thitipitchayanant conducted a study in Thailand and demonstrated that women with baby blues in whom relaxation methods were used (listening to mp3 recordings three times a day) had much lower levels of allopregnanolone. However, its high levels were associated with depression, stress and anxiety disorders when compared to the control group [45]. A study conducted by Akbarzadeh et al. revealed that women who attended religious meetings weekly during pregnancy less commonly reported the symptoms of baby blues compared to the control group [46]. Religion is deemed to be a preventive factor in mood disorders leading to the improvement of mental health, interpersonal relations and reduction of anxiety. According to the authors, mothers who received social support in their environment were more satisfied with being a mother. Encouraging to perform physical activity is another form of support. Susukida et al. reported that patients engaged in more physical activity during pregnancy were at a lower risk of psychological distress and postpartum depression [47].

### 4.1. Strengths of the Study

The present authors would like to emphasize the significant advantages of the paper:

1. The paper is a result of the analysis of material obtained in the course of reviewing medical records of a large group of women (604 individuals).
2. The assessment of the risk of developing postpartum mood disorders was performed with the use of a widely known and most commonly used tool in this aspect, i.e., Edinburgh Postnatal Depression Scale (EPDS), which makes the result comparable with the results obtained by other authors.
3. A high number of analyzed case histories facilitated the selection of two groups (the study and control group). Therefore, it was possible to perform comparative analyses.

### 4.2. Limitations of the Study

The limitations of the study are mostly due to its retrospective nature. They include the inability to identify factors which might indicate the correlation between the mental status of the patients directly during the test (situational factors) and inability to obtain the history directly from the patient which might be useful in distinguishing a group of patients with the symptoms of mental disease intragestationally. Notably, the occurrence of PPD depended on the cut-off value of the study. A Portuguese study, in which an analogous Edinburgh Postnatal Depression Scale (EPDS) was used, revealed that the percentage of reported PPD cases depended on the cut-off value and was 27.5% for scores >9 points and 14.2% for scores >12 points [48]. The cut-off value for patients with the risk of baby blues was relatively high (12 points) in the present study. In the control group it was low at 5 points. The liberalization of criteria would probably have an effect on the results. Moreover, the inclusion criteria for the control group also constituted a limitation, as they prevented the demonstration of correlations between age, marital status and education with the risk of postpartum depressive disorders. Moreover there is a high risk of false negatives of depression in anxiety patients [49]. The study does not describe medications used during delivery, which might have influenced the results as the conditions give rise to a higher risk of premature delivery. A correlation between those factors could not be demonstrated, as the number of such cases was low, which made it impossible to conduct suitable analyses.

## 5. Conclusions

The study revealed a relatively low percentage of women in labor with EPDS scores indicating a higher risk of developing depressive disorders after delivery (12.4%). Primigravidas and women who delivered prematurely were the most predisposed to developing postpartum depression. Primigravidas should undergo screening tests and psychological training to strengthen their sense of competence as a mother. Such interventions should be introduced over the perinatal period, e.g., during antenatal classes. Mothers who delivered prematurely belong to a group which should be offered more attention postnatally in order to provide them with adequate emotional and social support. The study also indicated the necessity of obtaining precise medical history, e.g., in an indirect form (a psychological test), in countries where mental disorders are socially stigmatized.

## Figures and Tables

**Table 1:** Demographic characteristics of study participants (*N*=150).

Variable	*N*	%
**Education**		
Primary or vocational	7	4.7%
Secondary	43	28.9%
Tertiary	100	66.4%
**Marital Status**		
Married	124	83%
Unmarried	23	15%
Divorced	3	2%
**Place of Residence**		
City	82	54.7%
Town	38	25.3%
Village	30	2%

**Table 2:** The comparison of variables associated with the course of delivery in the participants (the Mann–Whitney U test) (Z- Standardized Test Statistic).

	EPDS	N	Mean	Standard Deviation	Z	*p*
Gestational week	high	75	37.49	4.476	−2.896	0.004
	low	75	39.21	1.891		
Gravidity	high	75	1.55	0.874	−2.175	0.03
	low	75	1.85	0.982		
Parity	high	75	1.40	0.771	−1.948	0.05
	low	75	1,59	0.773		

**Table 3:** The comparison of the prevalence of premature rupture of membranes (PROM) in the participants (chi-square test).

			EDPS		Chi-Square	*p*
			High	Low		
PROM	0	N	25	32	0.499	0.480
		%	58.1%	65.3%		
	1	N	18	17		
		%	41.9%	34.7%		

**Table 4:** Diseases reported in the neonates (chi-square test).

			EDPS		Chi-Square	*p*
			High	Low		
Diseases and defects	No diseases or congenital defects	N	42	52	4.177	0.124
		%	56%	69.3%		
	A congenital defect in the neonate	N	12	5		
		%	16%	6.7%		
	A disease in the neonate (no congenital defects)	N	21	18		
		%	28%	24%		

**Table 5:** The comparison of tobacco smoking habits in the participants.

			EDPS		Z	*p*
			High	Low		
Smoking	No	N	64	68	1.72	0.190
		%	90.1%	95.8%		
	Yes	N	7	3		
		%	9.9%	4.2%		
